# Role of dimensional crossover on spin-orbit torque efficiency in magnetic insulator thin films

**DOI:** 10.1038/s41467-018-06059-7

**Published:** 2018-09-06

**Authors:** Qiming Shao, Chi Tang, Guoqiang Yu, Aryan Navabi, Hao Wu, Congli He, Junxue Li, Pramey Upadhyaya, Peng Zhang, Seyed Armin Razavi, Qing Lin He, Yawen Liu, Pei Yang, Se Kwon Kim, Cheng Zheng, Yizhou Liu, Lei Pan, Roger K. Lake, Xiufeng Han, Yaroslav Tserkovnyak, Jing Shi, Kang L. Wang

**Affiliations:** 1 Department of Electrical and Computer Engineering, University of California, Los Angeles, CA 90095 USA; 2 Department of Physics & Astronomy, University of California, Riverside, CA 92521 USA; 3 Beijing National Laboratory for Condensed Matter Physics, Institute of Physics, Chinese Academy of Sciences, Beijing, 100190 China; 4 Department of Physics & Astronomy, University of California, Los Angeles, CA 90095 USA; 5 York-Nanjing Joint Center for Spintronics and Nano Engineering (YNJC), School of Electronics Science and Engineering, Nanjing University, Nanjing, 210093 China; 6 Department of Electrical and Computer Engineering, University of California, Riverside, CA 92521 USA

## Abstract

Magnetic insulators (MIs) attract tremendous interest for spintronic applications due to low Gilbert damping and the absence of Ohmic loss. Spin-orbit torques (SOTs) on MIs are more intriguing than magnetic metals since SOTs cannot be transferred to MIs through direct injection of electron spins. Understanding of SOTs on MIs remains elusive, especially how SOTs scale with the MI film thickness. Here, we observe the critical role of dimensionality on the SOT efficiency by studying the MI layer thickness-dependent SOT efficiency in tungsten/thulium iron garnet (W/TmIG) bilayers. We show that the TmIG thin film evolves from two-dimensional to three-dimensional magnetic phase transitions as the thickness increases. We report the significant enhancement of the measured SOT efficiency as the TmIG thickness increases, which is attributed to the increase of the magnetic moment density. We demonstrate the current-induced SOT switching in the W/TmIG bilayers with a TmIG thickness up to 15 nm.

## Abstract (web summary)

The spin-orbit torque (SOT) induced magnetic switching makes metal/magnetic insulators bilayers preferred in the energy efficient spintronic applications. Here the authors show SOT switching in W/TmIG bilayers and reveal the dimension crossover of SOT as a function of TmIG thickness.

## Introduction

The interplay between heavy metals (HMs) and magnetic insulators (MIs) in heavy metal/magnetic insulator (HM/MI) bilayer systems has attracted tremendous attention from both fundamental research and practical applications^1–4^. First, the HM/MI bilayer benefits from the low Gilbert damping in the MI. In contrast to magnetic metal, MIs only allow spin information to propagate through magnons, instead of itinerant electrons, due to their large electronic bandgaps. The absence of Ohmic loss from the magnetic layer makes HM/MI bilayers more energy efficient than HM/magnetic metal bilayers.

The second advantage of the HM/MI bilayer is that the spin-orbit coupling in the HM or at the HM/MI interface allows the efficient generation of spin-orbit torques (SOTs) on the MI layer through the spin Hall effect (SHE) or Rashba–Edelstein effect^5–9^. These SOTs enable efficient manipulation of magnetization dynamics in the MI layer. Although the MI layer is electrically insulating, SOT-driven magnetization dynamics of MIs can be detected through anomalous Hall resistance (AHR) and spin Hall magnetoresistance (SMR) in the HM layer^10–13^. By probing the AHR, current-induced magnetization switching (CIMS) was observed in both Pt/BaFe_12_O_19_^14^ and Pt/Tm_3_Fe_5_O_12_ (TmIG) bilayers^15,16^. However, whether SOTs in Pt/MI bilayers are from SHE remains ambiguous due to the potential existence of the Rashba–Edelstein effect^16^. It remains unclear whether the switching direction will be opposite when we utilize HMs with opposite spin Hall angles. Moreover, the observed damping-like SOT efficiency (*ξ*_DL_) in the Pt/TmIG that is responsible for switching is still much lower than those in the Pt/ferromagnetic metals (FMs)^15,17,18^. To understand the origin of SOTs and to increase the value of *ξ*_DL_ in HM/MI bilayers, we utilize a HM with a large spin Hall angle opposite to that of Pt in a HM/MI bilayer, demonstrate magnetization switching, and analyze the contributions to the SOT.

In this article, we study the *ξ*_DL_ and CIMS in tungsten (W)/TmIG heterostructures with different TmIG layer thicknesses (*t*_TmIG_). The thickness dependence of the damping-like SOT allows us to understand the interplay between spin current and magnetism in TmIG. Here, W is chosen since it is reported to give the largest spin Hall angle among elemental HMs and its sign is opposite to that of Pt^19^. When the TmIG film thickness is reduced from 15 to 3.2 nm, the effective exchange coupling is strongly reduced due to long-wavelength thermal fluctuations, resulting in a dimensional crossover from three-dimension-like to two-dimension-like magnetic phase transitions. We quantify *ξ*_DL_ by using second-harmonic Hall measurements^20,21^. The *ξ*_DL_ increases with the *t*_TmIG_ in W/TmIG bilayers; this is attributed to the enhanced magnetic moment density due to suppression of thermal fluctuations. We then demonstrate the CIMS in W/TmIG bilayers up to *t*_TmIG_ = 15 nm; for *t*_TmIG_ = 15 nm, the switching current density is as low as 8 × 10^10^ A/m^2^. The estimated current switching efficiency enhances as *t*_TmIG_ increases, which is consistent with the increase of *ξ*_DL_ with *t*_TmIG_. Importantly, the switching direction of our W/TmIG devices is indeed opposite to that of the Pt/TmIG device^15^; this contrast confirms the important role of SHE in CIMS of MIs.

## Results

### Dimensional crossover of magnetism

**Fig. 1:**
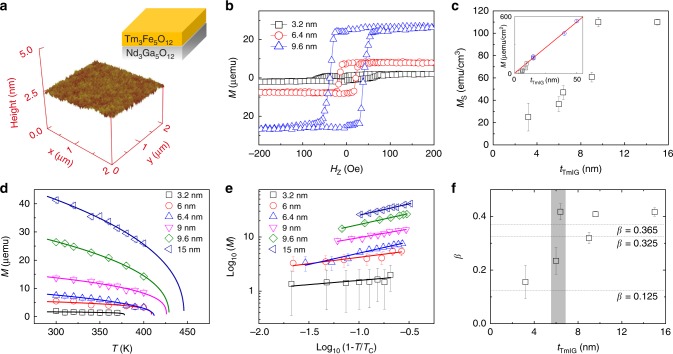
Dimensional crossover of magnetism in TmIG thin films. **a** Atomic force microscopy image of a 10 nm-thick TmIG film. **b** Magnetic moment as a function of out-of-plane magnetic field for TmIG thin films with different thicknesses at room temperature. **c** Saturation magnetization as a function of TmIG thickness at room temperature. The inset shows the areal magnetic moment as a function of TmIG thickness, which indicates a negligible magnetic dead layer. **d** Total magnetic moment as a function of temperature for different TmIG thicknesses. The solid lines are power-law fits to *M* = *M*_0_(1 − *T*/*T*_C_)^*β*^. **e** log_10_ (*M*) vs log_10_ (1 − *T*/*T*_C_) plots from (**d**) showing the thickness dependence of the *β* values. **f** Critical exponent *β* vs TmIG thickness showing a dimensional crossover from 2D to 3D. The dashed lines are theoretical values for 2D Ising (*β* = 0.125), 3D Ising (*β* = 0.325), and 3D Heisenberg (*β* = 0.365) models. The error bars in (**c**–**e**) stand for the measurement uncertainty, and the error bar in (**f**) stands for the fitting uncertainty

To access SOT and realize CIMS, we prepare high-quality TmIG thin films with different *t*_TmIG_ and characterize their magnetic properties. These TmIG(111) thin films were grown on substrate Nd_3_Ga_5_O_12_(111) by pulsed laser deposition^13^. All TmIG thin films show an atomically flat surface with mean roughness as low as 0.1 nm (Fig. 1a), providing a sharp interface for efficient spin momentum transfer. The Gilbert damping of TmIG thin films increases as the thickness decreases (see Supplementary Note 1). The large lattice mismatch between the TmIG and the Nd_3_Ga_5_O_12_ provides the tensile strain to generate perpendicular magnetic anisotropy in all TmIG thin films. The nature of perpendicular magnetic anisotropy is confirmed using magnetization hysteresis loops of TmIG thin films as a function of an out-of-plane magnetic field (Fig. 1b), from which we can determine saturation magnetization (*M*_S_). We observe a strong *t*_TmIG_ dependence of the *M*_S_ at room temperature (Fig. 1c); the *M*_S_ reduces significantly from the bulk *M*_S_ (110 emu/cm^3^)^22^ with decreasing film thickness. Note that the estimated dead layer thickness is less than 1 nm (see Fig. 1c inset and Supplementary Note 2), which also suggests a sharp interface between TmIG and substrate^23^. The reduction of the *M*_S_ at room temperature is attributed to finite size effect, strong thermal fluctuation and strong surface modification effect in ultrathin magnetic films^24–26^. Following ref. ^25^, we extract the critical exponents *β* for magnetic phase transitions in these TmIG thin films using temperature dependence of magnetic moment (*M*–*T*). The *M*–*T* curves follow the *M* = *M*_0_(1 − *T*/*T*_C_)^*β*^ (Fig. 1d), where zero-temperature magnetic moment (*M*_0_) and Curie temperature (*T*_C_) are fitting parameters. The *t*_TmIG_-dependent *β* is better illustrated using log–log plots as shown in Fig. 1e and the results are summarized in Fig. 1f. We see a clear increase of *β* from 0.16 ± 0.06 to 0.42 ± 0.02 when the *t*_TmIG_ increases from 3.2 to 15 nm, where the uncertainty is coming from the fitting. This increase of *β* suggests a dimensional crossover from two-dimension-like to three-dimension-like magnetism since 2D Ising model and 3D Heisenberg model predict *β* to be 0.125 and 0.365, respectively^26,27^. The dimensional crossover happens at around 6 nm, which is one order of magnitude larger than the typical transition thickness around 1 nm for magnetic metals^25–27^. In the following sections, we point out that the reduction of *M*_S_ due to dimensional crossover has a major influence on the magnitude of the SOT and switching efficiency, which has been neglected in the previous experiments.

### SOT measurement

**Fig. 2:**
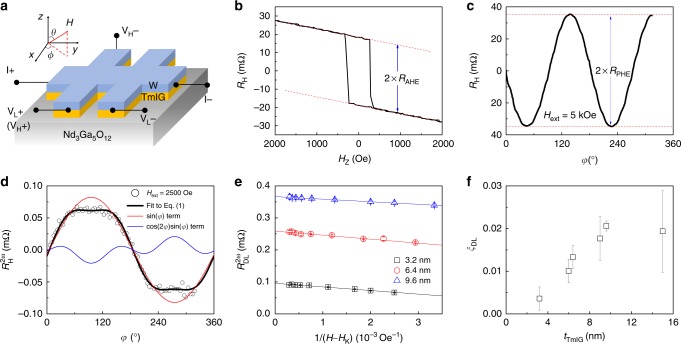
Spin transport and SOT measurements in the W/TmIG bilayers. **a** Experimental setup for measuring resistance, SOT, and current-induced magnetization switching. **b** Hall resistance as a function of an out-of-plane magnetic field for the W (5 nm)/TmIG (9.6 nm), where AHE is observed as the sharp square hysteresis loop. **c** Hall resistance as a function of a rotating in-plane constant magnetic field (5 kOe) for the W (5 nm)/TmIG (9.6 nm), where SMR-induced PHE is observed. **d** Second-harmonic Hall resistance as a function of in-plane azimuthal angle for the external magnetic field 2500 Oe for the W (5 nm)/TmIG (3.2 nm), where the black curve is the fit to Eq. (1). Both cos 2*φ*sin *φ* (blue curve) and sin *φ* (red curve) angle dependencies are revealed. **e** Extracted damping-like torque contribution as a function of the inverse of external magnetic field subtracting the anisotropy field. The large intercepts are the spin Seebeck resistance. **f** Damping-like spin-orbit torque efficiency as a function of TmIG thickness. The error bar stands for the fitting uncertainty

To perform resistance, SOT, and CIMS measurements, we fabricate W(5 nm)/TmIG(*t*_TmIG_) thin films into Hall bar devices (Fig. 2a). By using four-probe resistance measurements in different Hall bar devices, we determine the W resistivity to be 155 ± 15 µΩ·cm, where the uncertainty is estimated from the multiple (>20) device measurements. According to ref. ^19^, pure α-W has resistivity around 20 µΩ·cm, and 6 nm-thick W with mixed α- and β-phases has a resistivity as high as 170 µΩ·cm. So, most likely, our 5 nm-thick W thin films have mixed α- and β-phases. The AHR in the W/TmIG is accurately determined by the sharp anomalous Hall hysteresis at low fields (Fig. 2b). The transverse planar Hall resistance (PHR) accompanying the longitudinal SMR is measured by rotating the magnetization in the *xy*-plane (Fig. 2c). The observation of sizeable AHR and PHR (SMR) indicates that there is a significant spin current being transmitted across the W/TmIG interface or a sizable spin mixing conductance^11^ (see Supplementary Note 3).

We quantify *ξ*_DL_ by using the second-harmonic analysis of both AHR and PHR (*R*_AHE_ and *R*_PHE_)^20,21^. The second-harmonic Hall resistance ($R_{\mathrm{H}}^{2\omega }$) in a single domain subjected to an in-plane magnetic field can be written as^21,28^

1 $$R_{\mathrm{H}}^{2\omega } = \hskip1.5pt R_{{\mathrm{FL}}}^{2\omega }\cos 2\varphi \sin \varphi + R_{{\mathrm{DL}}}^{2\omega }\sin \varphi = R_{{\mathrm{PHE}}}\frac{{H_{{\mathrm{FL}}}}}{{\left| {H_{{\mathrm{ext}}}} \right|}}\cos 2\varphi \sin \varphi \\ + \left( {\frac{{R_{{\mathrm{AHE}}}}}{2}\frac{{H_{{\mathrm{DL}}}}}{{\left| {H_{{\mathrm{ext}}}} \right| - H_{\mathrm{K}}}} + R_{{\mathrm{SSE}}}} \right)\sin \varphi$$

where *H*_K_ and *H*_ext_ are perpendicular magnetic anisotropy effective field and in-plane external field, respectively. In Eq. (1), $R_{{\mathrm{FL}}}^{2\omega }$ and $R_{{\mathrm{DL}}}^{2\omega }$ are the peak values of cos 2*φ*sin *φ* and sin *φ* components in $R_{\mathrm{H}}^{2\omega }$, which are field-like SOT and damping-like SOT contributions, respectively. *H*_FL_ and *H*_DL_ are the current-induced field-like and damping-like effective fields, respectively. For example, when the *H*_ext_ = 2500 Oe, we observe significant contributions from both damping-like and field-like SOTs, as reflected by the cos 2*φ*sin *φ* and sin *φ* angle dependencies (see Fig. 2d and Supplementary Note 4). According to Eq. (1), slopes of linear fits to the $R_{{\mathrm{DL}}}^{2\omega }$ as a function of 1/(*H*_ext_ − *H*_K_) (Fig. 2e) give the information about *H*_DL_, and the intercepts are the spin Seebeck resistances (or voltages), which is field-independent in the single domain case (see Eq. (1))^21,29^.

We calculate *ξ*_DL_ using $\xi _{{\mathrm{DL}}} = \frac{{2eM_{\mathrm{S}}t_{{\mathrm{TmIG}}}H_{{\mathrm{DL}}}}}{{\hbar J_{{\mathrm{ac}}}}}$^6^, where *e* is the electron charge, *ħ* is the reduced Planck constant, and *J*_ac_ is the applied current density. We observe a characteristic increase of *ξ*_DL_ as *t*_TmIG_ increases with a saturation length of 10 nm (see Fig. 2f). Similarly, previous experiments have revealed a saturation length around 1 nm in FM heterostructures^18,30,31^. This saturation length is very close to the measured penetration depth of transverse spin current for FMs using spin pumping technique^32–34^. Thus, the saturation length has been interpreted as an indicator of penetration depth^33,34^. However, for our MI TmIG thin films, the scenario becomes complex since the electron spin cannot directly tunnel into the MI and the magnetism of MI thin films is strongly dependent on the MI thickness (Fig. 1). Note that the SOT efficiency (*ξ*_DL_~0.02) in our W/TmIG (≥9 nm) devices is smaller than that in β-W/CoFeB (ξ_DL_~0.3)^19^. There are two possible reasons. First, our W thin films are in mixed phases, which have a smaller spin Hall angle. Second, the material interfaces in W/magnetic metal and W/MI bilayers could be very different^17^, which requires further investigations.

### SOT switching

**Fig. 3:**
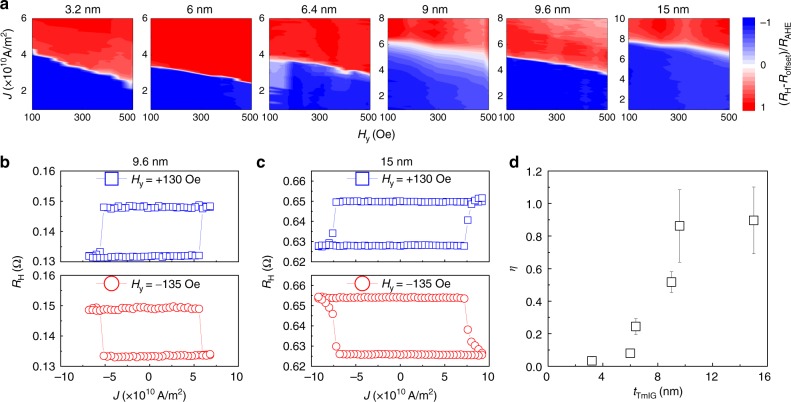
Current-induced magnetization switching in W/TmIG with different TmIG thicknesses. **a** Switching phase diagram for TmIG with thicknesses from 3.2 to 15 nm, where the external field is along the current direction. *R*_offset_ is device-dependent Hall resistance offset. For instances, **b**, **c** show the current-induced switching for TmIG with thickness 9.6 and 15 nm, respectively, in the presence of a magnetic field along and against the current direction. The switching is done by applying a 5 ms pulse with varying current amplitude. **d** TmIG thickness-dependent current switching efficiency, which is estimated from the depinning (coercive) field over switching current density in the zero-external field limit. The error bar originates from the multiple (>3) device measurements

After quantifying the SOT efficiency, we perform the CIMS experiments for W/TmIGs with different *t*_TmIG_. The switching is achieved in all devices with *t*_TmIG_ up to 15 nm and the switching phase diagrams are summarized in Fig. 3a. In the presence of an external field along the +*y* direction, a sufficiently large charge current along the +*y* direction will cause magnetization (AHR) switching from the +*z* direction to the −*z* direction (negative to positive). The required amount of charge current to flip the magnetization decreases as the external field increases. When we apply a sufficiently large charge current along the −*y* direction while keeping the external field along the +*y* direction, the magnetization (AHR) is switched from the −*z* direction to the +*z* direction (positive to negative) (upper panels in Fig. 3b, c). For the same current direction, the switching direction is opposite when we reverse the external field direction (lower panels in Fig. 3b, c). All of the above facts agree with the picture of SOT-driven magnetization switching. Note that the switching current density is as low as 6 × 10^10^ A/m^2^ for the W (5 nm)/TmIG (9.6 nm) (Fig. 3b), which is three times smaller than the Pt (5 nm)/TmIG (8 nm) case^15^. This suggests that W enables more energy efficient magnetization switching.

The switching direction driven by current-induced SOTs is consistent with the sign of the spin Hall angle of W, and it is opposite to that in the Pt/TmIG bilayer^15^. Therefore, our work strongly suggests the dominant role of the SHE in the generation of SOTs and CIMS in HM/MI bilayers. However, we do notice that there could be an interfacial Rashba–Edelstein effect at the W/TmIG interface contributing to the SOTs by comparative analyses of SOTs and SMR (AHR) (see Supplementary Note 5).

To quantitatively compare the switching efficiency of W/TmIG devices with different *t*_TmIG_, we define an effective switching efficiency as $\eta = \frac{{2eM_{\mathrm{S}}t_{{\mathrm{TmIG}}}H_{\mathrm{P}}}}{{\hbar J_{{\mathrm{sw}}}(H_{\mathrm{y}} \to 0)}}$^35^, where *H*_P_ is the domain wall depinning field estimated from the coercive field (see Supplementary Note 6) and *J*_sw_(*H*_y_ → 0) is the zero-field limit of current density in the switching phase diagram. This formula is chosen because the CIMS is achieved through domain nucleation and domain wall motion in the Hall bar devices due to the large scale of our Hall bar devices, of which the channel width is 20 µm^36^. We observe a dramatic increase of *η* with *t*_TmIG_ (Fig. 3d), for which we consider two reasons. First, the *ξ*_DL_ increases with *t*_TmIG_, which means that the same amount of charge current in the W layer generates stronger damping-like SOT on the TmIG layer. Thus, the increase of *ξ*_DL_ contributes to a lower *J*_sw_ and thus a larger *η*. Second, the Joule heating effect becomes much more significant when a larger charge current is applied, which is the case for switching a thicker TmIG. Joule heating causes reduction of thermal stability through decreasing the *M*_S_ and *H*_P_; these two values will be smaller than those measured at the low current limit. Therefore, the *M*_S_ and *H*_P_ used to calculate *η* are overestimated, leading to a larger *η*.

## Discussion

Here, we discuss the mechanism for the MI thickness dependence of *ξ*_DL_. We propose that *ξ*_DL_ depends on *M*_S_ when *M*_S_ of the thin films is well below the corresponding bulk value. The Landau–Lifshitz–Gilbert equation in the presence of damping-like SOT can be written as

2 $$M_{\mathrm{S}} t_{\mathrm{M}} \frac{{\mathrm{d}} {\hat m}} {\mathrm{dt}} = - \gamma M_{\mathrm{S}}t_{\mathrm{M}} \hat m \times \vec H_{{\mathrm{eff}}} + \alpha M_{\mathrm{S}}t_{\mathrm{M}}\hat m \times \frac{{\mathrm{d}}{\hat m}} {{\mathrm{d}t}} \\ + \gamma J_{\mathrm{C}}\xi _{{\mathrm{DL}}}\frac{\hbar }{{2e}}\left( {\hat m \times \hat \sigma \times \hat m} \right)$$

where $\hat m$ is the unit vector of magnetization, $\hat \sigma$ is the unit vector of current-induced spin polarization, *γ* is the gyromagnetic ratio, *α* is the Gilbert damping, *t*_M_ is the thickness of the magnetic layer, *J*_C_ is the charge current density, and $\vec H_{{\mathrm{eff}}}( = \vec H_{\mathrm{K}} + \vec H_{{\mathrm{ext}}})$ is the total effective magnetic field acting on the magnetization. The last term on the right-hand side of Eq. (2) arises due to the absorption of transverse spin current by the magnet, which is referred to as the current-induced damping-like (dissipative) SOT. Its strength is parameterized by dimensionless efficiency parameters *ξ*_DL_. The origin of the SOT can be understood in a simple microscopic picture as follows. A charge current at the HM and ferromagnet interface induces an accumulation of spin density, $\rho \hat \sigma$, due to the finite spin-orbit interaction (for example, by SHE or Rashba–Edelstein effect). Here, *ρ* is the magnitude of the spin density, which is proportional to the strength of the spin-orbit interaction. This spin density interacts with the ferromagnet via exchange interaction, of the form $U_{{\mathrm{ex}}} \sim \rho M_{\mathrm{S}}\hat m \cdot \hat \sigma$, enabling the absorption of the spin current by the ferromagnet. In the perturbative treatment, the spin current absorbed by the ferromagnet can be obtained up to second order in the exchange interaction to yield the damping-like SOT with $\xi _{{\mathrm{DL}}}\sim M_{\mathrm{S}}^2$^37^. The positive correlation between *ξ*_DL_ and *M*_S_ is referred as the *M*_S_-effect; it has also been theoretically studied in the frame of spin pumping effect (in Appendix B of ref. ^38^), which is the Onsager reciprocal process of the spin torque effect. The increase of spin mixing conductance with *M*_S_ is consistent with the calculation from first principles^39^ when the surface modification effect presents in the ultrathin regime^26^.

**Fig. 4:**
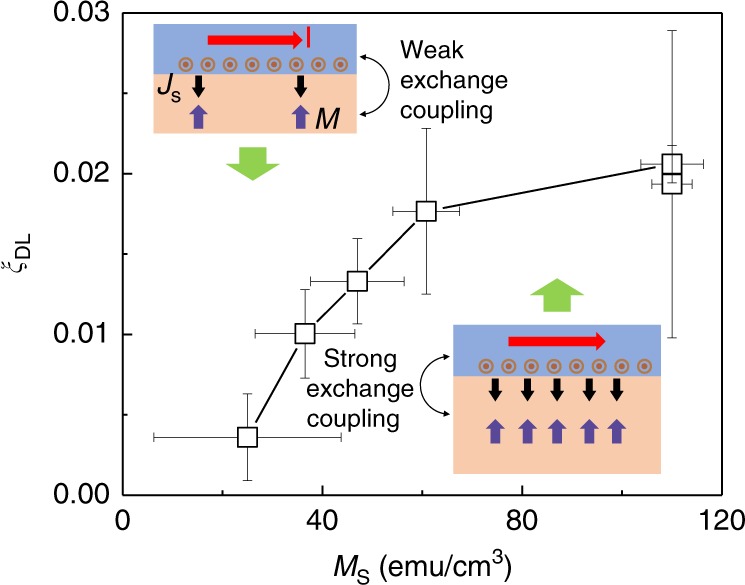
Role of TmIG *M*_S_ on the *ξ*_DL_. *ξ*_DL_ is proportional to the *M*_S_ squared as shown in the text when the *M*_S_ is small due to strong thermal fluctuation and surface modification effect. Insets show two cases: in the left inset, the magnetic moment density is small and thus the interfacial exchange interaction is weak, resulting in a small spin current injection; in the right inset, the magnetic moment density is large due to suppressed thermal fluctuation and thus the interfacial exchange interaction is strong, resulting in a large spin current injection. Definitions of the error bars for *M*_S_ and *ξ*_DL_ are given in Figs. 1, 2, respectively

Our experiments are the demonstrations of the *M*_S_-effect; we show that as the thickness increases, the SOT efficiency significantly increases with *M*_S_ in the low *M*_S_-regime (see Fig. 4), which is in qualitative agreement with the *M*_S_-effect. Also, we show that as the temperature decreases, the SOT efficiency increases with *M*_S_, due to suppression of thermal fluctuations (see Supplementary Note 7). Intuitively, as the magnetic moment density (*M*_S_) increases, the interfacial exchange interaction is enhanced, which allows more spin current to pass through the interface. As the thickness increases, the SOT efficiency saturates earlier than *M*_S_, around half of the bulk magnetization (60 emu/cm^3^), which suggests that the SOT is determined by the local magnetization that is saturated at a smaller thickness than the global magnetization *M*_S_. Our experiments show the need for further investigation of the interaction between ultrathin magnetic films and HMs, which would include the spin physics of dimensional crossover.

In summary, we have systematically studied the dimensional crossover of magnetism and its effect on SOTs in ultrathin MI films with perpendicular magnetic anisotropy. The characteristic increase of SOT efficiency with the MI thickness can be understood from the enhancement of magnetic moment density and the suppression of thermal fluctuations. In addition, we have realized CIMS in W/TmIG devices with *t*_TmIG_ up to 15 nm. The switching current density for W/TmIG devices is lower or comparable with these for HM/FM despite the fact that the saturated *ξ*_DL_ is estimated to be only around 0.02 at this stage, which is much less than the 0.3 that is estimated for W in W/CoFeB bilayers^19^. Further improvement of the *ξ*_DL_ could be done by spin mixing conductance matching^40^ and surface treatment^41^. Our results presented here show the great potential of ultrathin MI-based spintronics.

## Methods

### Materials growth and characterization

All TmIG(111) films were grown on Nd_3_Ga_5_O_12_(111) by pulsed laser deposition^13^ before transferring to a magnetron sputtering chamber in the ambient condition. At room temperature, we deposited a 5 nm-thick W layer on top of TmIG followed by subsequent deposition of MgO (2 nm)/TaO_*x*_ (3 nm) layers to protect W from oxidization. Magnetization hysteresis loops as a function of an out-of-plane magnetic field were measured by a vibrating sample magnetometer and a superconducting quantum interference device. The nominal thin film area is 5 × 5 mm^2^.

### Devices fabrication and characterization

The films were patterned into Hall bar devices (Fig. 2a) by using standard photolithography and dry etching for the resistance, SOT, and switching measurements. The channel width is 20 µm, and the distance between two neighboring Hall contacts is 26 µm. We measured the second-harmonic Hall resistance by applying *I*_ac,r.m.s_ = 1 mA (*J*_ac,r.m.s_ = 10^10^ A/m^2^) with a frequency *ω*/2*π* = 195.85 Hz. The magnetic field and angle controls were done in a physical properties measurement system. The CIMS experiments were performed in the ambient environment by applying a pulse current with 5 ms pulse width and reading Hall voltage subsequently.

## Electronic supplementary material

Supplementary Information

## Data Availability

The data that support the findings of this study are available from the corresponding authors upon reasonable request.

## References

[1] Kajiwara Y (2010). Transmission of electrical signals by spin-wave interconversion in a magnetic insulator. Nature.

[2] Bauer GE, Saitoh E, van Wees BJ (2012). Spin caloritronics. Nat. Mater..

[3] Uchida K (2008). Observation of the spin Seebeck effect. Nature.

[4] Wu, M.-Z. & Hoffmann, A. *Recent Advances in Magnetic Insulators—From Spintronics to Microwave Applications* (Academic Press, 2013).

[5] Miron IM (2011). Perpendicular switching of a single ferromagnetic layer induced by in-plane current injection. Nature.

[6] Liu L (2012). Spin-torque switching with the giant spin Hall effect of tantalum. Science.

[7] Qiu X (2015). Spin-orbit-torque engineering via oxygen manipulation. Nat. Nanotechnol..

[8] Yu G (2014). Switching of perpendicular magnetization by spin-orbit torques in the absence of external magnetic fields. Nat. Nanotechol..

[9] Liu L, Lee OJ, Gudmundsen TJ, Ralph DC, Buhrman RA (2012). Current-induced switching of perpendicularly magnetized magnetic layers using spin torque from the spin Hall effect. Phys. Rev. Lett..

[10] Nakayama H (2013). Spin Hall magnetoresistance induced by a nonequilibrium proximity effect. Phys. Rev. Lett..

[11] Chen YT (2013). Theory of spin Hall magnetoresistance. Phys. Rev. B.

[12] Hahn C (2013). Comparative measurements of inverse spin Hall effects and magnetoresistance in YIG/Pt and YIG/Ta. Phys. Rev. B.

[13] Tang C (2016). Anomalous Hall hysteresis in Tm_3_Fe_5_O_12_/Pt with strain-induced perpendicular magnetic anisotropy. Phys. Rev. B.

[14] Li P (2016). Spin-orbit torque-assisted switching in magnetic insulator thin films with perpendicular magnetic anisotropy. Nat. Commun..

[15] Avci CO (2017). Current-induced switching in a magnetic insulator. Nat. Mater..

[16] Li J (2017). Deficiency of the bulk spin Hall effect model for spin-orbit torques in magnetic-insulator/heavy-metal heterostructures. Phys. Rev. B.

[17] Zhang W, Han W, Jiang X, Yang SH, Parkin SSP (2015). Role of transparency of platinum–ferromagnet interfaces in determining the intrinsic magnitude of the spin Hall effect. Nat. Phys..

[18] Pai CF, Ou Y, Vilela-Leão LH, Ralph DC, Buhrman RA (2015). Dependence of the efficiency of spin Hall torque on the transparency of Pt/ferromagnetic layer interfaces. Phys. Rev. B.

[19] Pai CF (2012). Spin transfer torque devices utilizing the giant spin Hall effect of tungsten. Appl. Phys. Lett..

[20] Garello K (2013). Symmetry and magnitude of spin-orbit torques in ferromagnetic heterostructures. Nat. Nanotechnol..

[21] Avci CO (2014). Interplay of spin-orbit torque and thermoelectric effects in ferromagnet/normal-metal bilayers. Phys. Rev. B.

[22] Paoletti, A. *Physics of Magnetic Garnets* (North-Holland Publishing Company, 1978).

[23] Tang C (2017). Above 400-K robust perpendicular ferromagnetic phase in a topological insulator. Sci. Adv..

[24] Zhang R, Willis RF (2001). Thickness-dependent Curie temperatures of ultrathin magnetic films: effect of the range of spin-spin interactions. Phys. Rev. Lett..

[25] Huang F, Kief MT, Mankey GJ, Willis RF (1994). Magnetism in the few-monolayers limit: a surface magneto-optic Kerr-effect study of the magnetic behavior of ultrathin films of Co, Ni, and Co–Ni alloys on Cu(100) and Cu(111). Phys. Rev. B.

[26] Vaz CAF, Bland JAC, Lauhoff G (2008). Magnetism in ultrathin film structures. Rep. Prog. Phys..

[27] Li Y, Baberschke K (1992). Dimensional crossover in ultrathin Ni(111) films on W(110). Phys. Rev. Lett..

[28] Shao Q (2016). Strong Rashba–Edelstein effect-induced spin-orbit torques in monolayer transition metal dichalcogenide/ferromagnet bilayers. Nano Lett..

[29] Uchida Ki (2010). Observation of longitudinal spin-Seebeck effect in magnetic insulators. Appl. Phys. Lett..

[30] Kim J (2013). Layer thickness dependence of the current-induced effective field vector in Ta|CoFeB|MgO. Nat. Mater..

[31] Pai CF, Mann M, Tan AJ, Beach GSD (2016). Determination of spin torque efficiencies in heterostructures with perpendicular magnetic anisotropy. Phys. Rev. B.

[32] Ghosh A, Auffret S, Ebels U, Bailey WE (2012). Penetration depth of transverse spin current in ultrathin ferromagnets. Phys. Rev. Lett..

[33] Qiu X (2016). Enhanced spin-orbit torque via modulation of spin current absorption. Phys. Rev. Lett..

[34] Ou Y, Pai CF, Shi S, Ralph DC, Buhrman RA (2016). Origin of fieldlike spin-orbit torques in heavy metal/ferromagnet/oxide thin film heterostructures. Phys. Rev. B.

[35] Lee OJ (2014). Central role of domain wall depinning for perpendicular magnetization switching driven by spin torque from the spin Hall effect. Phys. Rev. B.

[36] Yu G (2014). Magnetization switching through spin-Hall-effect-induced chiral domain wall propagation. Phys. Rev. B.

[37] Bender SA, Tserkovnyak Y (2015). Interfacial spin and heat transfer between metals and magnetic insulators. Phys. Rev. B.

[38] Ohnuma Y, Adachi H, Saitoh E, Maekawa S (2014). Enhanced dc spin pumping into a fluctuating ferromagnet near *T*_C_. Phys. Rev. B.

[39] Jia X, Liu K, Xia K, Bauer GEW (2011). Spin transfer torque on magnetic insulators. Europhys. Lett..

[40] Du C, Wang H, Yang F, Hammel PC (2014). Enhancement of pure spin currents in spin pumping Y_3_Fe_5_O_12_/Cu/metal trilayers through spin conductance matching. Phys. Rev. Appl..

[41] Jungfleisch MB, Lauer V, Neb R, Chumak AV, Hillebrands B (2013). Improvement of the yttrium iron garnet/platinum interface for spin pumping-based applications. Appl. Phys. Lett..

