# Supplementary material for: Role of dimensional crossover on spin-orbit torque efficiency in magnetic insulator thin films
Source: Nat Commun. 2018 Sep 6;9:3612. doi: 10.1038/s41467-018-06059-7 (PMC6127239; doi:10.1038/s41467-018-06059-7)
Supplement: Supplementary file 1 — Supplementary Information [file 41467_2018_6059_MOESM1_ESM.docx]

**Supplemental Information**

**Role of dimensional crossover on spin-orbit torque efficiency in magnetic insulator thin films**

**Shao *et al.***

**Supplementary Note 1. Thickness dependence of damping in TmIG thin films**

We use spin-torque ferromagnetic resonance to determine the damping constant of another series of TmIG thin films (Supplementary Figure 1). We see that the damping factor increases when the TmIG film thickness decreases, which is typical for magnetic thin films.

**Supplementary Figure 1**. Spin-torque ferromagnetic resonance characterization of thickness dependent damping factors. (a) Spectra of a 4.8 nm-thick TmIG. Red curves are fits to the resonant peaks ^1^. (b) Extracted linewidth of in-plane resonance peaks for different thicknesses. Solid curves are linear fits. The error bars originate from the fitting uncertainty. (c) Extracted damping factor as a function of the TmIG thickness.

**Supplementary Note 2. Discussions on the estimation of magnetic dead layer (MDL) thickness**

If we do a linear fit to thickness dependent magnetic moment per unit area *M* for thickness from 9.6 nm to 50 nm, we obtain a negligible MDL thickness (*t*_MDL_) around 0 nm (Supplementary Figure 2a). We can calculate *M*_S_ by using $M_{S}=M/\left( t-t_{\mathrm{MDL}} \right)$ and the results are shown in Supplementary Figure 2c, where the saturated value of *M*_S_ is around the bulk value 110 emu/cm^3^ from the literature. Alternatively, if we do a linear fit for thickness from 9 nm to 50 nm, we obtain a *t*_MDL_ around 1 nm (Supplementary Figure 2b). Correspondingly, the obtained thickness dependent *M*_S_ is shown in Supplementary Figure 2d, where values of *M*_S_ larger than 110 emu/cm^3^ are observed. This suggests that this 1 nm *t*_MDL_ is overestimated. Nevertheless, we still observe that the *M*_S_ increases dramatically as the TmIG thickness increases before the thickness reaches 10 nm, which is consistent with Fig. 1c and Supplementary Figure 2c. More importantly, the exact value of *t*_MDL_ will not affect our observations as follows. The dimensional crossover is observed using$M=M_{0}\left( 1-T/{T_{C}} \right)^{\beta}$, which describes the temperature dependence of magnetic moment and is irrelevant to the effective thickness (*t* - *t*_MDL_), The SOT efficiency ($\xi_{\mathrm{DL}}=\frac{2eMH_{\mathrm{DL}}}{\hbar J_{\mathrm{ac}}}$) and switching efficiency ($\eta=\frac{2eMH_{P}}{\hbar J_{\mathrm{sw}}}$) only depend on the *M*, which is the measured areal magnetization, independent of the effective thickness (*t* - *t*_MDL_).

**Supplementary Figure 2**. Thickness dependence of magnetic moment per unit area *M* and magnetic moment per unit volume *M*_S_ from different fitting ranges. Red curve is a linear fit to thickness ranging from 9.6 nm to 50 nm (a) and 9 nm to 50 nm (b). Black square symbols are films being used for SOT and switching studies. Blue circle symbols are additional films for determining magnetic dead layer. The estimated magnetic dead layer thickness is 0 nm for (a) and 1 nm for (b). (c) The calculated *M*_S_ a function of thickness for 0 nm magnetic dead layer. (d) The calculated *M*_S_ a function of thickness for 1 nm magnetic dead layer. The black dashed line in (c) and (d) is bulk magnetization value 110 emu/cm^3^ from literature ^2^. The error bars stand for the measurement uncertainty.

**Supplementary Note 3. Spin mixing conductance from the spin Hall magnetoresistance (SMR) measurement**

First, we assume a spin Hall effect picture for the W/TmIG. According to SMR theory ^3^, the spin mixing conductance ($G_{\uparrow\downarrow}=G_{r}+iG_{i}$) can be estimated by using $\frac{\rho_{\mathrm{SMR}}}{\rho}=\frac{2\theta_{\mathrm{SH}}^{2}\lambda^{2}}{d}\mathrm{Re}\frac{G_{\uparrow\downarrow}\tanh^{2} \frac{d}{2\lambda}}{\frac{1}{\rho}+2\lambda G_{\uparrow\downarrow}\coth\frac{d}{\lambda}}$ and $\frac{\rho_{SMR-AH}}{\rho}=-\frac{2\theta_{\mathrm{SH}}^{2}\lambda^{2}}{d}\mathrm{Im}\frac{G_{\uparrow\downarrow}\tanh^{2} \frac{d}{2\lambda}}{\frac{1}{\rho}+2\lambda G_{\uparrow\downarrow}\coth\frac{d}{\lambda}}$, where $\theta_{\mathrm{SH}}$ is the spin Hall angle, *d* is the W thickness, $\rho$ is the resistivity 155±15 µΩ·cm and $\lambda$ is the spin diffusion length of W layer, respectively. The magnitude of SMR is determined as shown in Supplementary Figure 3. If we assume the $\theta_{\mathrm{SH}}$ is 0.3 ^4^ and the $\lambda$ is 2.1 nm ^5^, we get $G_{r}\approx2\times{10}^{12}\Omega^{-1}m^{-2}$ and $G_{i}\approx1\times{10}^{12}\Omega^{-1}m^{-2}$.

**Supplementary Figure 3.** Longitudinal resistance as a function of an external magnetic field along the ±*x* direction for the W (5 nm)/TmIG (9.6 nm), where SMR is observed as the resistance is minimized when magnetization is along the ±*x* direction.

**Supplementary Note 4. Discussions on field-like torque**

In principle, we can determine the field-like SOT effective field *H*_FL_ using Eq. (1): $R_{H}^{2\omega}=R_{\mathrm{FL}}^{2\omega}\cos2\varphi\sin\varphi+R_{\mathrm{DL}}^{2\omega}\sin\varphi=R_{\mathrm{PHE}}\frac{H_{\mathrm{FL}}}{\left| H_{\mathrm{ext}} \right|}\cos2\varphi\sin\varphi+\left( \frac{R_{\mathrm{AHE}}}{2}\frac{H_{\mathrm{DL}}}{\left| H_{\mathrm{ext}} \right|-H_{K}}+R_{\mathrm{SSE}} \right)\sin\varphi$, where we can distinguish damping-like SOT (spin Seebeck effect) and field-like SOT contributions by their angle dependencies. As shown in Fig. 2d and discussed in the paragraph below Eq. (1) in the main text, we observed significant contribution from field-like SOT. According to Eq. (1), slopes of linear fits to the field-like SOT contribution $R_{\mathrm{FL}}^{2\omega}$ as a function of 1/*H*_K_ (Supplementary Figure 4a) give the information of *H*_FL_. Ideally, the intercepts should be close to zero. However, we do observe large intercepts that accompany the large error bars in the $R_{\mathrm{FL}}^{2\omega}$ of thicker TmIG devices (Supplementary Figure 4a). The reason for the nonzero intercepts could be as follows. As the $R_{\mathrm{FL}}^{2\omega}$ is divergent near zero field according to Eq. (1), the $R_{\mathrm{FL}}^{2\omega}$ decreases significantly and becomes very small in the range 1500 – 5000 Oe that we use to pull magnetization to the in-plane single domain state for determining SOT efficiency. As a result, the dominant contribution $R_{\mathrm{DL}}^{2\omega}$ will mix into the $\cos2\varphi\sin\varphi$ part, resulting into a large error bar and sizeable intercept of $R_{\mathrm{FL}}^{2\omega}$. Therefore, the quantitative determination of *H*_FL_ becomes difficult. Nevertheless, we can still estimate the field-like SOT efficiency by using $\xi_{\mathrm{FL}}=\frac{2eM_{S}t_{\mathrm{TmIG}}\left( H_{\mathrm{FL}}+H_{\mathrm{Oersted}} \right)}{\hbar J_{\mathrm{ac}}}$ (Supplementary Figure 4b). Note that the current-induced Oersted field has been considered since it has the same symmetry as the field-like SOT effective field.

**Supplementary Figure 4**. (a) Extracted field-like torque contribution ($R_{\mathrm{FL}}^{2\omega}$) as a function of the inverse of external magnetic field. The intercepts are due to the uncertainty during the extraction of $R_{\mathrm{FL}}^{2\omega}$, where the large signals with $\sin\varphi$ angle dependence come in ^6^. (b) Field-like SOT efficiency as a function of TmIG thickness. The error bars originate from the fitting uncertainty.

**Supplementary Note 5. Possible Rashba-Edelstein effect at the interface**

Here, we present the inconsistency of determined spin transparency $T_{r}$ between SMR and $\xi_{\mathrm{DL}}$. After we obtaining $G_{\uparrow\downarrow}$ using SMR as done in Section S1, we can calculate the spin transparency following $T_{r}=Re\frac{2G_{\uparrow\downarrow}\tanh\frac{d}{2\lambda}}{\frac{1}{\rho\lambda}+2G_{\uparrow\downarrow}\coth\frac{d}{\lambda}}$ ^3^. On the other hand, the $T_{r}$ can also be directly determined using $T_{r}=\frac{\xi_{\mathrm{DL}}}{\theta_{\mathrm{SH}}}$ ^3,6^. In Supplementary Figure 5, we plot the TmIG thickness dependent $T_{r}$ estimated from $\xi_{\mathrm{DL}}$ and SMR with different $\lambda$’s. The quantitative difference suggests the complex relation between spin current effect and interfacial spin transparency. A good match with extremely small (and unrealistic) $\lambda$ highlights the importance of interfacial effects, such as Rashba-Edelstein effect. Note that another interfacial effect, magnetic proximity effect, is not considered since the W is far away from the Stoner instability.

**Supplementary Figure 5**. Values of $T_{r}$ estimated from $\xi_{\mathrm{DL}}$ and SMR. We assume the spin Hall angle is 0.3 ^2^ and the spin diffusion length varies from 0.2 nm to 2.1 nm ^5,8^.

**Supplementary Note 6. Thickness dependence of PMA effective field and coercive field**

**Supplementary Figure 6**. TmIG thickness dependent PMA effective field (a) and coercive field (b) at room temperature. The error bar in (b) is from the variation of coercive fields in different Hall bar devices. The error bars are estimated from the multiple (> 6) device measurements.

**Supplementary Note 7. Temperature dependence of magnetization and damping-like SOT efficiency**

We show the temperature dependence of *M*_S_ for three samples, 3.2 nm-, 6.4 nm- and 9.6 nm-thick TmIG thin films (Supplementary Figure 7a), which were measured before the Hall bar device fabrication. Indeed, the difference between *M*_S_’s of these three samples at low temperature (250 K) is slightly smaller than those at high temperatures (300 – 350 K).

We also performed second harmonic measurements at different temperatures to determine the temperature dependence of damping-like SOT efficiency $R_{\mathrm{FL}}^{2\omega}$. We apply external field along the $\varphi=45^{\circ}$ direction while we measure the second harmonic Hall resistance. Now, Eq. (1) becomes $R_{H}^{2\omega}=\frac{\sqrt{2}}{2}\left( \frac{R_{\mathrm{AHE}}}{2}\frac{H_{\mathrm{DL}}}{\left| H_{\mathrm{ext}} \right|-H_{K}}+R_{\mathrm{SSE}} \right)$, which can be used to extract relevant damping-like SOT effective field *H*_DL_. Supplementary Figure 7b show the thickness dependent second harmonic Hall resistance together with fitting curves. From Supplementary Figure 7c, we observed that the difference between $R_{\mathrm{DL}}^{2\omega}$’s in these three samples is significantly smaller than those at high temperatures (300 - 350 K). Our observations of temperature dependence of *M*_S_ and $R_{\mathrm{DL}}^{2\omega}$ are qualitatively in agreement with the conclusion that the thermal fluctuations suppress the SOT efficiency.


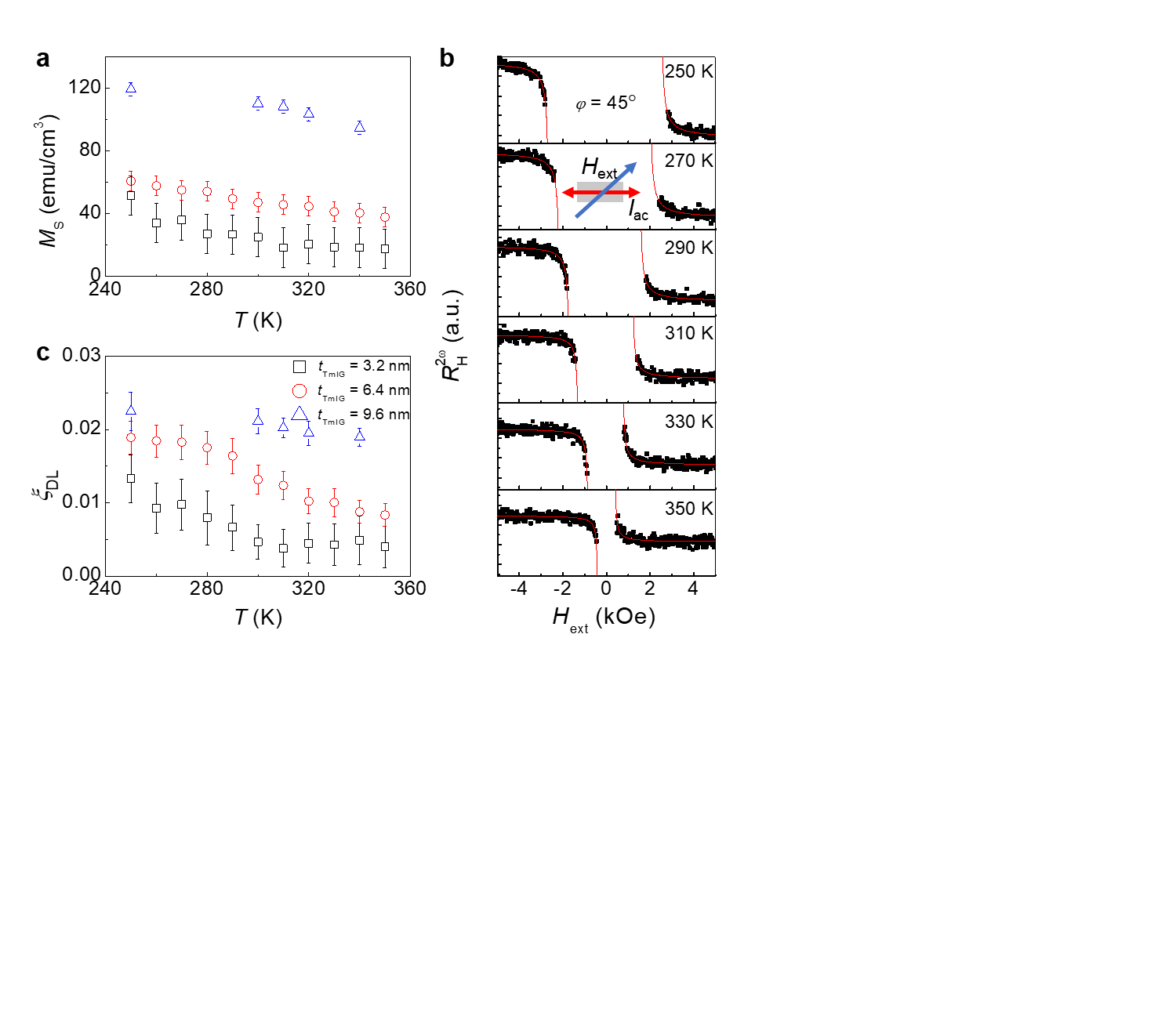


**Supplementary Figure 7**. Temperature dependence of saturation magnetization *M*_S_ (a) and damping-like SOT efficiency $R_{\mathrm{DL}}^{2\omega}$ (c) for different thicknesses. The error bars in (a) and (c) stand for the measurement uncertainty and fitting uncertainty, respectively. (b) Temperature dependence of second harmonic Hall resistance at different temperatures. The external field direction is $\varphi=45^{\circ}$ as shown in the inset of top second panel.

We would like to emphasize that any mechanism that affects the *M*_S_ would in principle affect the SOT efficiency. Our data about thickness and temperature dependence of *M*_S_ and SOT efficiency strongly suggest that the role of thermal fluctuations is important. However, we cannot fully exclude other effects, such as the surface modification effect mentioned in ref. ^9^, which shows that the surface modification effect in the ultrathin magnetic films could play an important role in determining the *M*_S_.

There is a technical challenge to get an accurate damping-like spin-orbit torque (SOT) efficiency $\xi_{\mathrm{DL}}$ at lower temperatures (below 250 K) for thick TmIG samples. As described by Eq. (1), the second-harmonic Hall resistance $R_{H}^{2\omega}$ is divergent at the anisotropy field ($H_{K}$) since $R_{H}^{2\omega}=\frac{\sqrt{2}}{2}\left( \frac{R_{\mathrm{AHE}}}{2}\frac{H_{\mathrm{DL}}}{\left| H_{\mathrm{ext}} \right|-H_{K}}+R_{\mathrm{SSE}} \right)$ when $\varphi=45^{\circ}$ and the external field is along the defined positive direction (Supplementary Figure 8). When $\varphi=45^{\circ}$ and the external field is along the defined negative direction, $R_{H}^{2\omega}=-\frac{\sqrt{2}}{2}\left( \frac{R_{\mathrm{AHE}}}{2}\frac{H_{\mathrm{DL}}}{\left| H_{\mathrm{ext}} \right|-H_{K}}+R_{\mathrm{SSE}} \right)$. Note that the contribution of spin Seebeck effect $R_{\mathrm{SSE}}$ is proportional to the in-plane magnetization ($m_{\mathrm{ip}}$), which is a smooth (non-divergent) function from +*M*_S_ to -*M*_S_ that saturates at the ${\pm H}_{K}$when the external field is swept from the large positive field to the large negative field. Therefore, the existence of the peaks due to damping-like SOT facilitates the accurate determination of the damping-like SOT effective field ($H_{\mathrm{DL}}$) by fitting the region where the external field is larger than the $H_{K}$. In this work, we determine the temperature dependence of $\xi_{\mathrm{DL}}$ between 250 K and 350 K for TmIG samples with thickness 3.2 nm, 6.4 nm and 9.6 nm, where the peaks due to the damping-like SOT are well observed (Supplementary Figure 8). As temperature decreases, the peaks become less clear (Supplementary Figure 8). This problem is more apparent when the TmIG thickness increases since given the same $\xi_{\mathrm{DL}}$, the larger TmIG thickness gives rise to a smaller $H_{\mathrm{DL}}$ according to $H_{\mathrm{DL}}=\frac{\xi_{\mathrm{DL}}\hbar J_{\mathrm{ac}}}{2eM_{s}t_{\mathrm{TmIG}}}$. By comparing the $R_{H}^{2\omega}$ for the W/TmIG(9.6 nm) and the W/TmIG (3.2 nm), we observe that the peaks due to the damping-like SOT are less clear in the W/TmIG(9.6 nm) at 250 K (Supplementary Figure 8a), but very clear in the W/TmIG(3.2 nm) at 250 K (Supplementary Figure 8b). Therefore, we cannot simply use the field-dependence of $R_{H}^{2\omega}$ to determine the $H_{\mathrm{DL}}$ in thicker TmIG films at lower temperatures, such as temperatures below 250 K.

**Supplementary Figure 8**. Second harmonic Hall resistance ($R_{H}^{2\omega}$) as a function of external in-plane magnetic field at different temperatures in the W/TmIG(9.6 nm) (a) and the W/TmIG(3.2 nm) (b), where $\varphi=45^{\circ}$. The difference in the saturated $R_{H}^{2\omega}$ under large positive and negative fields is due to the spin Seebeck effect ($R_{\mathrm{SSE}}$). The black arrows in (a) highlight the peaks due to damping-like spin-orbit torque.

**Supplementary References**

1 He, C. *et al.* Spin-torque ferromagnetic resonance measurements utilizing spin Hall magnetoresistance in W/Co_40_Fe_40_B_20_/MgO structures. *Appl. Phys. Lett.* **109**, 202404 (2016).

2 Paoletti, A. *Physics of Magnetic Garnets*. (North-Holland Publishing Company, 1978).

3 Chen, Y.-T. *et al.* Theory of spin Hall magnetoresistance. *Phys. Rev. B* **87**, 144411 (2013).

4 Pai, C.-F. *et al.* Spin transfer torque devices utilizing the giant spin Hall effect of tungsten. *Appl. Phys. Lett.* **101**, 122404 (2012).

5 Wang, H. L. *et al.* Scaling of spin Hall angle in 3d, 4d, and 5d metals from Y_3_Fe_5_O_12_/metal spin pumping. *Phys. Rev. Lett.* **112**, 197201 (2014).

6 Shao, Q. *et al.* Strong Rashba-Edelstein effect-induced spin-orbit torques in monolayer transition metal dichalcogenide/ferromagnet bilayers. *Nano Lett.* **16**, 7514 (2016).

7 Pai, C.-F., Mann, M., Tan, A. J. & Beach, G. S. D. Determination of spin torque efficiencies in heterostructures with perpendicular magnetic anisotropy. *Phys. Rev. B* **93**, 144409 (2016).

8 Kim, J., Sheng, P., Takahashi, S., Mitani, S. & Hayashi, M. Spin Hall Magnetoresistance in Metallic Bilayers. *Phys. Rev. Lett.* **116**, 097201 (2016).

9 Vaz, C. A. F., Bland, J. A. C. & Lauhoff, G. Magnetism in ultrathin film structures. *Rep. Prog. Phys.* **71**, 056501 (2008).
